# Transplants, Open Top Chambers (OTCs) and Gradient Studies Ask Different Questions in Climate Change Effects Studies

**DOI:** 10.3389/fpls.2018.01574

**Published:** 2018-11-02

**Authors:** Yan Yang, Aud H. Halbritter, Kari Klanderud, Richard J. Telford, Genxu Wang, Vigdis Vandvik

**Affiliations:** ^1^Institute of Mountain Hazards and Environment, Chinese Academy of Sciences, Chengdu, China; ^2^Department of Biological Sciences, University of Bergen, Bergen, Norway; ^3^Bjerknes Centre for Climate Research, University of Bergen, Bergen, Norway; ^4^Faculty of Environmental Sciences and Natural Resource Management, Norwegian University of Life Sciences, Ås, Norway

**Keywords:** alpine grasslands, experimental warming, integrated approaches, space-for-time, southwestern China

## Abstract

Long-term monitoring, space-for-time substitutions along gradients, and *in situ* temperature manipulations are common approaches to understand effects of climate change on alpine and arctic plant communities. Although general patterns emerge from studies using different approaches, there are also some inconsistencies. To provide better estimates of plant community responses to future warming across a range of environments, there have been repeated calls for integrating different approaches within single studies. Thus, to examine how different methods in climate change effect studies may ask different questions, we combined three climate warming approaches in a single study in the Hengduan Mountains of southwestern China. We monitored plant communities along an elevation gradient using the space-for-time approach, and conducted warming experiments using open top chambers (OTCs) and plant community transplantation toward warmer climates along the same gradient. Plant species richness and abundances were monitored over 5 years addressing two questions: (1) how do plant communities respond to the different climate warming approaches? (2) how can the combined approaches improve predictions of plant community responses to climate change? The general trend across all three approaches was decreased species richness with climate warming at low elevations. This suggests increased competition from immigrating lowland species, and/or from the species already growing inside the plots, as indicated by increased biomass, vegetation height or proportion of graminoids. At the coldest sites, species richness decreased in OTCs and along the gradient, but increased in the transplants, suggesting that plant communities in colder climates are more open to invasion from lowland species, with slow species loss. This was only detected in the transplants, showing that different approaches, may yield different results. Whereas OTCs may constrain immigration of new species, transplanted communities are rapidly exposed to new neighbors that can easily colonize the small plots. Thus, different approaches ask slightly different questions, in particular regarding indirect climate change effects, such as biotic interactions. To better understand both direct and indirect effects of climate change on plant communities, we need to combine approaches in future studies, and if novel interactions are of particular interest, transplants may be a better approach than OTCs.

## Introduction

Alpine ecosystems are temperature limited systems and have long been predicted to be sensitive to climate change (Walker et al., 2006; Elmendorf et al., 2015). The projected rate of future warming in the alpine region is also faster than the global average (IPCC, 2013). Thus, understanding how climate warming affects alpine plant communities is especially important for our ability to predict impacts of future climate change.

An increasing number of studies report that climate warming is driving essential changes in alpine and arctic plant communities, such as phenology (Wolkovich et al., 2012; Li et al., 2016), species distribution ranges (Van der Wal et al., 2012; Grytnes et al., 2014), species abundances (Elmendorf et al., 2012a,b) and species richness (Pauli et al., 2012; Wipf et al., 2013; Steinbauer et al., 2018). Although there are general patterns emerging, there are also some inconsistencies. For example, experimental warming by open top chambers (OTCs) in Tibet observed delayed reproductive phenology and decreased number of inflorescences of dominant species (Dorji et al., 2014), whereas warming by transplantation to lower elevations found advanced flowering dates (Wang et al., 2014). Furthermore, whereas warming by OTCs decrease alpine and arctic species diversity (Elmendorf et al., 2012a), studies resampling historical data find increased species richness in alpine regions (Steinbauer et al., 2018). Part of this variation can be due to different methods (Elmendorf et al., 2015), and typically four primary approaches have been used to study impacts of climate warming on alpine plant community properties: studies along natural elevation gradients (space-for-time substitution), resampling or monitoring over time, experimental warming by OTCs, and whole-community transplantation to warmer climates.

Elevation gradient studies allow investigating community responses to a broader range of both abiotic and biotic environmental conditions, including direct and indirect effects of climate change. These communities do, however, reflect responses over longer time scales, and may therefore overestimate plant community responses to current climate changes (Elmendorf et al., 2015). Resampling or long-term monitoring can provide important information on how plant communities respond to both short-term changes in weather and long-term changes in climate (Gottfried et al., 2012; Wipf et al., 2013). However, these approaches require historical data, or several years of monitoring, and, as both resampling and long-term monitoring are observational approaches, they cannot disentangle the different factors driving the changes observed. Experimental warming can better explore cause-and-effect relationships between plant communities and climate change, as well as provide a mechanistic understanding of short-term responses of ecosystems to climate warming (Rustad, 2006). *In situ* passive warming approaches, such as OTCs, have widely been applied to warm extant vegetation in alpine and arctic regions (Elmendorf et al., 2012a). The walls of the OTCs, may, however, constrain immigration and migration of plant species in the same way as they have been shown to affect pollination by wind and insects (Totland and Eide, 1999; Richardson et al., 2000). Thus, OTCs may not take into account novel interactions from new colonizers in warmer climates (Alexander et al., 2015). OTCs are also conservative warming devices, with an increase of mean daily temperature by *c.* 1.5°C in alpine and arctic tundra (Marion et al., 1997), which in some areas, is low compared to variations between years (Hollister and Webber, 2000). They therefore need to be installed for a long time to affect the vegetation (Hollister et al., 2005; Hudson and Henry, 2010). Indeed, high stability and resistance observed in low-productivity alpine and arctic plant communities to warming by OTCs can be due to the minor warming (Hudson and Henry, 2010; Keuper et al., 2011). Importantly, the warming effect of OTCs can be confounded with changes in the microenvironment such as soil moisture, wind or snow accumulation (e.g., Marion et al., 1997; Hollister et al., 2005). Another approach that contrasts the traditional *in situ* warming experiments is plant community transplantation (Nooten and Hughes, 2017), following classical transplants of individual plants (Clausen et al., 1948). Intact turfs of whole plant communities are moved to a lower elevation exposing it to a warmer climate in combination with a new neighborhood community. In other words, transplantation may change both the abiotic and the biotic environment, and can therefore examine the net effect of both direct and indirect impacts of climate warming (Alexander et al., 2015).

Thus, different approaches address specific questions in their own way (Elmendorf et al., 2015). To provide better estimates of plant community responses to future warming across a range of environments, there is a call for integrating different approaches within single studies (Dunne et al., 2004; Rustad, 2008; Menke et al., 2014). Here, we used two experimental warming approaches parallel and integrated them with a gradient approach in a single study in the Hengduan Mountains of southwest China to test congruency among them. We conducted warming experiments using *in situ* OTCs, and community transplantation along an elevation gradient, and we monitored control plots (space-for-time approach) over 5 years. Plant species richness and cover were measured each year from 2012 to 2016. Our study addresses two questions: (1) how do plant communities respond to the three different climate warming approaches? (2) how can the combined approaches improve predictions of plant community responses to climate change?

## Materials and Methods

### Study Area

The study was conducted in the Hengduan Mountains, in southwestern China. The study sites are located in Kang-Ding Valley, northwest of Mt. Gongga (Supplementary Figure S1), which is characterized by steep vertical elevational gradients, and vegetation belts changing from mixed coniferous-broadleaves forest and subalpine coniferous forest, to shrubs and alpine meadows with increasing elevation (Liu et al., 1985; Shen et al., 2001). Long term climate data extracted from Worldclim version 2.0 from the period 1970–2000 in the area show a mean annual temperature of 11.6°C and mean annual precipitation of c. 800 mm (Fick and Hijmans, 2017). We selected four perennial grassland sites spanning from the mixed leaf forest to the alpine climatic zone, differing on average by 1.75°C (range: 1.5–2.1°C ) mean air temperature (June–August) between the sites (5.3°C between the lowest to the highest site), including a Low (3000 m a.s.l.), Middle (3500 m a.s.l.), Alpine (3850 m a.s.l.), and High alpine (4130 m a.s.l.), site (Table 1 and Supplementary Figure S1). The geographical distance between adjacent sites is on average 10 km. The sites were selected to be as similar as possible in terms of vegetation, soil and grazing regime to enable between-site comparisons. The vegetation in all sites is dominated by grasses (*Festuca* spp., *Poa* spp.,), sedges (*Carex* spp., *Kobresia* spp.), forbs (e.g., *Anaphalis nepalensis, Clinopodium polycephalum, Geranium pylzowianum, Polygonum viviparum, Potentilla leuconata, P. stenophylla*, and *Saussurea* spp.), and some shrubs (*Rhododendron* spp.) at the highest sites (see Supplementary Table S2 for species list). All the sites are associated with mountain gray-brown soil originating from granite (He et al., 2005). There is moderate livestock grazing by yak, sheep, or horses in all the sites, and fences were used during the study to prevent grazers inside the plots. We simulated grazing by cutting vegetation to ca 5 cm to avoid any fence effects.

**Table 1:** The four study sites with elevation, geographical coordinates, summer mean temperature (June–August) measured at 2 m between 2012 and 2016, long term annual precipitation from Worldclim version 2.0 for 1970 – 2000 (Fick and Hijmans, 2017), soil moisture (June–August) measured at 5 cm below ground between 2012 and 2016 and productivity measured as biomass per 0.5 m × 0.5 m plots in the Hengduan Mountains, China.

Site	Elevation (m a.s.l.)	Latitude (°N)	Longitude (°E)	Summer mean temperature (°C)	Annual precipitation (mm)	Soil moisture (%)	Productivity g/0.5 m^2^
High alpine	4130	29.91	102.01	6.7	797	0.36	34.2 ± 2.2
Alpine	3850	29.89	102.02	8.4	821	0.38	62.2 ± 4.1
Middle	3500	29.86	102.04	9.9	775	0.46	89.2 ± 6.3
Low	3000	29.84	102.03	12.0	784	0.38	67.9 ± 4.4

### Experimental Design

Seven blocks were randomly positioned in each of the sites in 2012, covering an area of ca 400 m^2^. The distance between the replicate blocks ranges from 4 to 6 m. In each block, we randomly positioned four 25 cm × 25 cm plots. One was used for *in situ* warming by OTC, one was transplanted to warmer climates, one was transplanted locally within the same block to control for any transplant effect, and one was used as an untouched control (Supplementary Figure S1). Thus, the blocks provided seven replicates for each of the three approaches.

Analysis of the local transplant and the untouched control plots across all years show that there were no differences between them, and thus no unwanted effects of the turf cutting and transplanting, as also shown in a similar transplant experiment using the same approach (Guittar et al., 2016). For the gradient study, we therefore used both the local transplant and the untouched control plot as space-for-time substitutions along the elevation gradient with ca. 1.5°C temperature difference between each of the sites (Table 1).

The OTCs were placed upon one plot in each block, with the plot in the center. The OTCs are 40 cm tall, and the distance between parallel sides is 106 cm at the base and 60 cm at the top. Generally, OTCs increase mean daily air temperature by c. 1.5°C (Marion et al., 1997), which we could not test due to climate logger failures in our experiment and therefore refer to the expected warming in the literature. For the transplant experiment, one plot at each block per site was transplanted to the corresponding block of the site at the lower elevation with c. 1.75°C warmer summer temperature (i.e., temperature difference between each site). We permanently marked each corner of the plots with plastic poles. For the transplanted plots, the upslope center seen from the front of each turf was marked with a plastic flag, to ensure that the turfs were placed in the same position relative to the slope and block orientation at the target site. We used a knife to cut the transplanted plots 2 cm outside the margins, providing a buffer zone for possible edge effects, and at a depth of 20 cm, unless the soil was shallower, as was the case for some of the High alpine plots. After excavation, the plots were packed into boxes and transported to their respective target sites within 1 or 2 days. The turfs were fitted into the gaps created by excavating turfs at the target site and carefully checked that the soil surface was in plane with the surrounding vegetation, and that the edges of the excavated plot was in good contact with the edges of the gap. If necessary, loose soil was carefully removed from the underside of the turf, or local soil was added to the gap or around the edges to achieve this. In total there were 108 plots at the four sites along the elevational gradients, but four plots in the High alpine site were destroyed by yak in 2014.

### Data Collection

A climate station (U30-NRC, Onset, United States) at each site recorded air temperature at 2 m and soil moisture at −5 cm in 10 min intervals since September 2012 and during the whole study period. We measured biomass at each of the sites in 2015 to have an estimate of productivity along the gradient (Table 1). We did this by harvesting all above ground biomass from 13 0.5 m × 0.5 m plots at the High alpine site and 20 plots from the other sites, and oven-dried it at 60°C for 72 h before weighing.

All vascular plant species in each plot were surveyed in 2012 (before treatment), and annually between 2013 and 2016. Percent cover of each vascular species was visually estimated during the peak of the growing season using a 25 cm × 25 cm frame with a grid of 5 cm × 5 cm subplots. Mean vegetation height for each plot was measured at five points in 2013 using a ruler. Forbs were identified to species level, whereas many of the graminoids were identified to genus level, i.e., *Carex* spp., *Poa* spp., *Kobresia* spp., and *Festuca* spp., because of a lack of a detailed flora from the study region and thus difficulties with identification of sterile graminoids.

### Data Curation and Statistical Analyses

Over the 5 years of collecting this extensive data set, different people were involved, which increases the risk of observation errors. In particular, species can be misidentified (i.e., sterile graminoids) or might be overlooked in one of the observations. These errors will result in pseudo-turnover in the plant community data. To detect such errors, we compared each recorded species in each subplot over the 5 years. We used the subplots to assign unidentified or missing species if there was a record of the species in the previous and following year. Further, we re-estimated species cover in cases where cover was either too low or high to be real when comparing with other years, and replaced these values with the mean cover from the previous and following year. We did such re-estimates in totally 48 occasions (*c.* 1%) of the whole 5 years dataset.

To test how plant community properties change along the elevational gradient (i.e., represented as change in mean summer temperature among sites) and/or respond to the two different warming treatments (i.e., mean summer temperature contrasts between control and warming treatment), we fit linear models independently for each warming approach with species richness, evenness, or proportion of graminoids as response variable. We checked the fulfillment of the model assumptions visually and evenness had a heavy tail of negative residuals (2–3 observations). For the gradient approach, we fit two models: an intercept-only null model (no effect) and a model with mean summer temperature at each site (effect). For the two experimental warming treatments, we fitted three models: one containing the experimental site (no effect), one with experimental site and temperature contrast (effect) and one with the interactions of experimental site and temperature contrast (interaction). The effects of experimental site and temperature were tested by comparing the difference in Aikaike information criterion (AIC) score Δ_i_ among these models differing in their fixed effects (Burnham and Anderson, 2002). A difference in AIC scores of Δ_i_ > 2 between models indicates strong support for the model with lower AIC score (Burnham and Anderson, 2002). The model with the lowest AIC score was chosen as the best model, except when competing models differed by Δ_i_ < 2, then the model with fewest parameters was selected.

To quantify and visualize the temporal changes in species composition between the warming approaches, we used principle response curves (PRC; Van den Brink and Ter Braak, 1999). PRC is the multivariate equivalent of repeated measures ANOVA, and analyses the community response through time to one or more treatments relative to a control. It is a partial RDA where treatments and time are included as factorial variables in a model analyzing the effects of the time x treatment interaction while including time as a covariate to control for any overall temporal trends. Treatment effects (*C*_dt_) quantify the compositional difference between treated plots and controls at each sampling date, and temporal trends can be visualized by plotting *C*_dt_ against time. We performed two separate PRCs on the forb community only because of the more detailed taxonomic resolution in the forbs. Rare species, that occurred less than three times in the whole data set were removed for this analysis.

First, we quantified how much the species communities responded to the two warming approaches by moving “*away*” from the origin control communities (i.e., how much does the species community in the OTC or the transplant at the High alpine site differ from the control communities at the High alpine site). For this, we performed a PRC using species cover from 2012 to 2016 from the controls at the origin site and the two warming treatments. The analysis was done separately for each site and the two warming treatments. To test if the communities in the warming approaches differed from the origin controls communities, we used a permutation test. We used the species scores to assess and compare the species responses to the individual treatments.

Second, we tested if the species communities in the two warming treatments moved “toward” the target communities (i.e., to what extent does species community in the OTC at the High alpine site and the community transplanted from the High alpine to the Alpine site become similar to the target control communities at the Alpine site). We used a similar PRC approach as above, but run one model for each origin site with both warming approaches and the target control communities as treatments. The result was visualized by plotting the treatment effects *C*_dt_ for each of these treatments against time.

All analyses were performed in R 3.4.4 (R Core Team, 2018) using the vegan packages (Oksanen et al., 2018). All data and R code will be made available at OSF repository (doi:10.17605/OSF.IO/F3KN, https://osf.io/f3knq/).

## Results

Temperature measurements show that mean annual and summer temperatures increase along the gradient from the high to the low elevation sites by ca 1.75°C between each site (Table 1). Biomass increased toward lower elevations along the gradient, with a peak in the Middle site (*F*_3,69_ = 19.02, *P* < 0.001; Table 1). Vegetation height after 1 year of treatment increased in the OTCs compared to the control plots by 3.01 (SE 0.19) cm in the High alpine, 4.21 (SE 0.26) cm in the Alpine, 8.58 (SE 0.89) cm in the Middle, and 8.22 (SE 1.13) cm in the Low elevation site (*F*_1,54_ = 5.75, *P* = 0.02).

**FIGURE 1:**
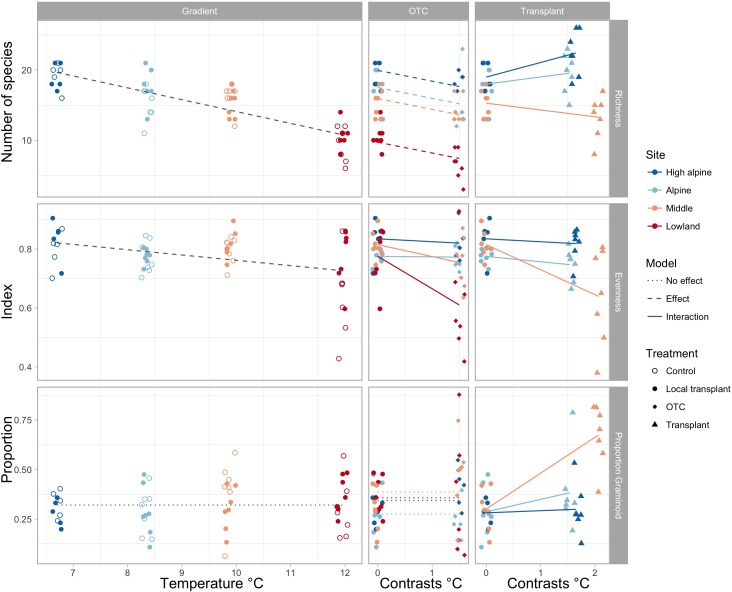
Change in species richness, evenness, and proportion of graminoids along the elevation gradient, represented by mean summer temperature **(left)**, and in OTCs and transplants, represented by 1.5 and 1.75°C temperature contrasts **(right)**. Significant interactions of warming effects between sites are shown by solid lines, significant overall effects across sites in stapled lines, and no effects in dotted lines. Different colors indicate the sites, High alpine, Alpine, Middle and Lowland, and symbols show the treatments, control, local transplant, OTC and transplant.

Species richness gradually decreases toward warmer climates, both in the ambient plots along the elevation gradient and in the OTCs (Figure 1 and Supplementary Tables S3, S4). Transplanting to warmer climates appears to increase species richness in the coldest sites, but decrease species richness in the warmest site. Evenness decreased in OTCs at the warmest site and in plots transplanted to a lower elevation in the warmest climates, i.e., from the Middle to the Low elevation site. The proportion of graminoids increased toward warmer climates only in plots transplanted to the warmest elevation (Figure 1 and Supplementary Tables S3, S4).

**FIGURE 2:**
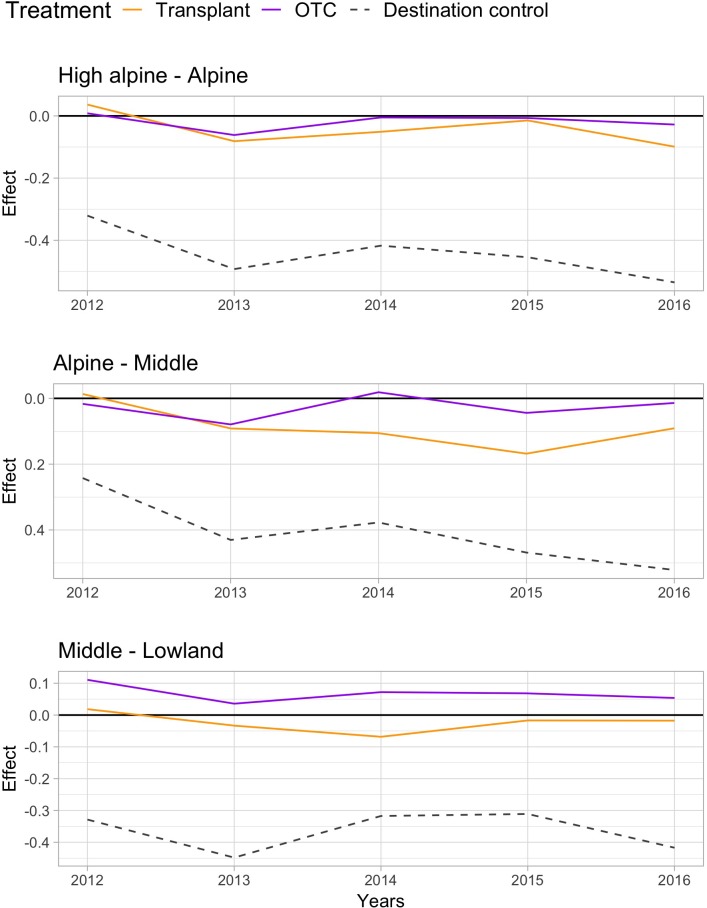
Principle response curves (PRC) diagrams and species scores on PRC axis 1, showing the overall impact of the two warming treatments OTC and transplant and the destination control on species composition (treatment effects; PRC axis 1 or *C*_dt_) at the High alpine – Alpine **(top)**, Alpine – Middle **(middle)**, and Middle – Lowland site **(bottom)**. Negative values indicate a community shifts “toward” the target community, positive values the opposite way, and values near zero indicate no response to the treatment.

Warming by both OTCs and transplants changed the species communities over the 5 years “*away*” from the control communities at the origin sites, with the change being stronger at lower elevations (Table 2). Species composition in the OTC responded less to warming along the elevation gradient compared to the transplanted communities, i.e., they differed significantly stronger from the control communities at the Middle site. Interestingly, species composition in the OTCs and in plots that have been transplanted to warmer climates seem to change in slightly different directions. Transplanted plots appear to move more consistently “*toward”* the target community, i.e., the species composition in the control plot of the site in which they have been transplanted to (Figure 2, orange line moves more toward the gray dashed line). More species showed a stronger positive or negative response the warming treatment in the transplants, i.e., *Persicaria vivipara*, *Saussurea ceterach* at the High alpine and Alpine site and *Pedicularis davidii* at the Alpine and Middle site (Supplementary Table S5). Across all sites, both the transplants and OTC communities were far away from converging toward the target community even after 5 years. And indeed, at the Middle site the OTC showed no indication of moving toward the target community.

**Table 2:** Results of the PRC showing the proportion of explained variation by the treatment and treatment × time interaction for each site.

Origin site	OTC	Transplant
High alpine	0.079	0.072
Alpine	0.078	**0.077^∗^**
Middle	**0.107^∗∗^**	**0.094^∗∗^**
Lowland	0.072	-

*Separate models for each warming treatment were used. Numbers in bold and with asterisk show significant differences in plant communities between the warming treatment and origin control. Bold values and asterisk indicate that the treatment differs significant from the origin control community. ^∗^p < 0.05, p^∗∗^ < 0.01*.

## Discussion

Here we combined three climate warming approaches in a single study and find similar responses in terms of plant community properties, with some exceptions. The general trend, in the elevation gradient and both experimental warming approaches, was decreased species richness and evenness with climate warming in the warmest sites at low elevation. This is in line with previous studies, suggesting increased competitive effects from the taller vegetation canopy of the species already growing in the plots and/or from lowland immigrating species, under warmer climates (e.g., Elmendorf et al., 2012a,b; Alexander et al., 2015). In our study, increased intensity of competition is indicated by the taller vegetation in the OTCs along the whole gradient, and the increase in proportions of graminoids in plots transplanted to warmer climates in the lowest elevations. The decrease in species richness and evenness observed toward warmer and lower elevation sites along the gradient in our study is also likely due to increased competition, as biomass increase, and the plants are generally bigger toward warmer sites, and thus fewer individuals and hence fewer species can fit into the plots.

In the colder climates at the higher elevations, on the other hand, plant community responses depended on the warming approach. In the transplants, species richness appeared to increase in plots transplanted toward warmer climates from the High alpine to the Alpine, and from the Alpine to the Middle site. This suggests that alpine vegetation is open to invasion by species from lower elevation, confirming the range shift literature that lowland species are moving to higher elevations (e.g., Klanderud and Birks, 2003; Gottfried et al., 2012; Pauli et al., 2012; Steinbauer et al., 2018). Higher invasibility of alpine plant communities has also been shown in a transplant experiment in Norway, where seedling recruitment was higher in alpine vegetation compared to vegetation at lower elevations (Meineri et al., unpublished). Moreover, species appear to have migrated into the transplanted plots in our study from the new neighborhood plant community, but without immediately outcompeting the species inhabiting the plots. Thus, it appears that alpine species are resistant to invasion and increased competitive effects in the short term, and that loss of species is a slow process in these cold environments. This is in line with a similar transplant experiment in alpine Norway (Vandvik et al., unpublished), and with resampling and long-term monitoring studies, showing that migration by lowland species to alpine sites is faster than the extinctions of the alpine specialists, resulting in increased species richness at high elevations, at least in the short term (e.g., Klanderud and Birks, 2003; Gottfried et al., 2012; Pauli et al., 2012; Steinbauer et al., 2018). Contrastingly, warming by OTCs decreased species richness across all sites in our study, which agrees with previous studies using OTCs in alpine and arctic tundra (e.g., Walker et al., 2006; Elmendorf et al., 2012a). This decrease in species richness is previously explained by increased competition inside the plots when the height of the vegetation canopy increase (Klanderud and Totland, 2005; Walker et al., 2006; Elmendorf et al., 2012a). Another possible explanation, in addition to competition, can be that new species have difficulties with entering the plots inside the OTCs because the walls may act as migration barriers. In addition, immigration of new species is likely lower in OTCs than in transplants in our study because of the longer distance to source populations. This can probably also explain the steep increase of graminoids in plots transplanted to the lowest elevation site, but no effects on graminoids in the OTCs. Transplants from colder climates are rapidly exposed to lowland graminoids that can enter the plots. For the plots in the OTCs on the other hand, it might take more time before graminoids already present inside or outside the OTCs increase in abundance or enter the plots. Thus, transplants are likely better means than OTCs in detecting effects of species migrations and novel interactions due to climate change (Suttle et al., 2007; Blois et al., 2013; Alexander et al., 2015).

Previous explanations of, in some cases, lack of responses of alpine and arctic plant communities to warming by OTCs have been that the OTCs only provide minor warming in comparison to year-to-year variability (Hollister and Webber, 2000; Hudson and Henry, 2010; Keuper et al., 2011). The lack of warming by OTCs during nights, and higher maximum temperatures during the day (Marion et al., 1997; Klein et al., 2005; Sierra-Almeida and Cavieres, 2010; Godfree et al., 2011), also results in larger diurnal ranges in the OTCs than in the transplants and in the communities along the natural gradient in our study. Drought stress induced by high temperatures and, in some cases slightly (not statistical significant) lower soil moisture inside OTCs (Bokhorst et al., 2013), may decrease alpine plant species richness (Klein et al., 2004). We don’t think, however, that this explains the decrease in species richness inside OTCs in our study, as humidity is relatively high in this area, and the plots are positioned on slopes where the water moves easily through the soil. Our study shows that plant communities respond slightly differently to approximately the same degree of mean summer temperature increase, although the warming effect of the OTCs was likely lower than the other approaches also in our study. This suggests, however, that other factors than temperature *per se*, such as species ability to migrate in and out of the plots, and species interactions, may explain the different responses of the plant communities between the different approaches in our study. Other factors not measured in our study, such as soil properties, herbivory, microclimate, or pathogens may also have affected our results, but we did not observe any signs of this in the field.

Novel interactions from lowland species that are tracking climate warming and hence immigrating into the alpine communities are likely to be important for the performance of alpine species in the future (Alexander et al., 2015). The approaches studied in this paper differ in their ability to mimic, such indirect impacts of climate warming on plant communities. Whereas OTCs may act as barriers to species migration, transplanting plots to a new environment provide a very rapid exposure to new potential colonizing species. The realistic future scenario is probably somewhere between the recruitment limitations by the OTCs and the extreme exposure to new neighbors in the transplants. The elevation gradient approach reflects long-term responses to species migrations and biotic interactions, and over much longer time scales than can be explained by short-term experiments (Elmendorf et al., 2015). However, slowly changing plant communities along spatial temperature gradients may lag behind the more rapid anthropogenic climate change (Pickett, 1989). Combining and integrating more than one approach is likely the best tool to examine responses of both direct warming effects, and indirect effects of changes in biotic interactions due to species migrations. Our results show, however, that the choice of approach depends on the research question of the particular project. If the aim is to understand not only direct effects, but also indirect effects of climate warming on plant communities, such as biotic interactions, transplantations will probably examine the role of novel species interactions in a better way than OTCs.

## Author Contributions

YY secured funding for the project, ran the experiment, and wrote the manuscript. AH analyzed data and wrote the manuscript. KK designed the experiment, conceived the idea of this paper, and wrote the manuscript. RT prepared and analyzed the data and commented on the manuscript. GW secured funding for the project and IMHE research infrastructure and commented on the manuscript. VV designed the experiment, conceived the idea of this paper, and commented on the manuscript.

## Conflict of Interest Statement

The authors declare that the research was conducted in the absence of any commercial or financial relationships that could be construed as a potential conflict of interest.
