# Supplementary material for: Transplants, Open Top Chambers (OTCs) and Gradient Studies Ask Different Questions in Climate Change Effects Studies
Source: Front Plant Sci. 2018 Nov 2;9:1574. doi: 10.3389/fpls.2018.01574 (PMC6224372; doi:10.3389/fpls.2018.01574)
Supplement: Supplementary file 1 [file Data_Sheet_1.docx]

|  | 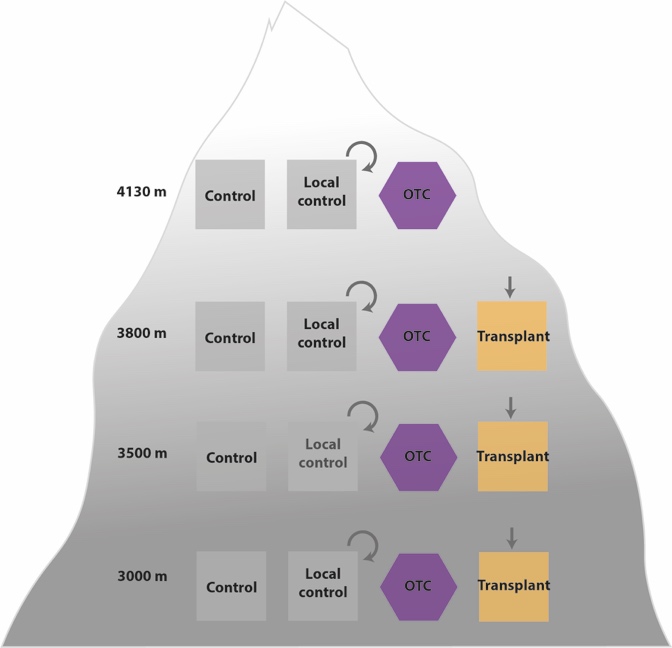 |
| --- | --- |
| **Figure S1** Location of the four study sites along an elevational gradient in the Hengduan mountains in China (left). Inset indicates the location of the study sites in China. Experimental set up for the different warming treatments at each site. Shown are control, local control, OTC and transplant and arrows indicate direction of transplant. | |

| **Table S2** Functional group and occurrence of species at the four experimental sites: High Alpine, Alpine, Middle and Lowland. | | | | | |
| --- | --- | --- | --- | --- | --- |
| **Functional Group** | **Species** | **High Alpine** | **Alpine** | **Middle** | **Lowland** |
| Forb | *Acanthocalyx nepalensis* | x | - | x | - |
| Forb | *Ajuga decumbens* | - | - | - | x |
| Forb | *Aletris pauciflora* | x | x | - | - |
| Forb | *Allium prattii* | x | x | - | - |
| Forb | *Allium sikkimense* | - | x | - | - |
| Forb | *Anaphalis aureopunctata* | x | x | x | x |
| Forb | *Anaphalis flavescens* | x | x | x | - |
| Forb | *Androsace minor* | x | x | x | - |
| Forb | *Anemone demissa* | x | x | - | - |
| Forb | *Anemone rivularis* | - | - | - | x |
| Forb | *Arisaema parvum* | - | x | x | - |
| Forb | *Artemisia flaccida* | x | x | x | - |
| Forb | *Aster asteroides* | x | x | - | x |
| Forb | *Athyrium subtriangulare* | - | x | x | - |
| Forb | *Caltha palustris* | x | x | - | - |
| Forb | *Carpesium humile* | - | x | x | - |
| Forb | *Cerastium szechuense* | x | x | x | x |
| Forb | *Chamaesium viridiflorum* | x | - | - | - |
| Forb | *Clematis pseudopogonandra* | - | - | x | - |
| Forb | *Clinopodium polycephalum* | x | x | x | x |
| Forb | *Codonopsis foetens* | x | x | - | - |
| Forb | *Cyananthus incanus* | x | x | x | - |
| Forb | *Epilobium fangii* | x | x | x | x |
| Forb | *Euphorbia spp.* | x | - | - | - |
| Forb | *Fragaria orientalis* | x | x | x | x |
| Forb | *Galearis spathulata* | x | x | - | - |
| Forb | *Galium spp* | - | x | x | x |
| Forb | *Gentiana crassuloides* | x | x | x | - |
| Forb | *Gentiana trichotoma* | x | - | - | - |
| Forb | *Geranium nepalense* | - | - | - | x |
| Forb | *Geranium pylzowianum* | x | x | x | x |
| Forb | *Geum aleppicum* | - | - | x | x |
| Forb | *Gymnadenia conopsea* | x | - | - | - |
| Forb | *Halenia corniculata* | x | x | x | x |
| Forb | *Hedysarum algidum* | x | x | - | - |
| Forb | *Hemiphragma heterophyllum* | x | x | x | x |
| Forb | *Hypericum wightianum* | - | x | x | x |
| Forb | *Ligularia pleurocaulis* | - | x | - | - |
| Forb | *Ligusticum scapiforme* | x | x | x | - |
| Forb | *Lilium lophophorum* | - | - | x | - |
| Forb | *Lomatogonium macranthum* | x | - | - | - |
| Forb | *Maianthemum henryi* | - | - | x | - |
| Forb | *Maianthemum japonicum* | x | x | x | - |
| Forb | *Malaxis monophyllos* | x | x | x | - |
| Forb | *Microula sikkimensis* | - | - | x | - |
| Forb | *Morina nepalensis* | x | - | - | - |
| Forb | *Myriactis nepalensis* | - | - | - | x |
| Forb | *Notholirion bulbuliferum* | - | - | x | - |
| Forb | *Oxytropis yunnanensis* | x | x | - | - |
| Forb | *Parasenecio latipes* | x | x | x | - |
| Forb | *Parnassia spp* | x | - | - | - |
| Forb | *Pedicularis davidii* | - | x | x | - |
| Forb | *Pedicularis mussotii var. lophocentra* | - | x | - | - |
| Forb | *Pedicularis rhynchodonta* | x | - | - | - |
| Forb | *Pedicularis sima* | x | x | x | - |
| Forb | *Persicaria vivipara* | x | x | x | - |
| Forb | *Phlomoides medicinalis* | - | - | x | - |
| Forb | *Plantago asiatica* | x | x | x | x |
| Forb | *Polygonatum cirrhifolium* | - | x | x | - |
| Forb | *Polygonum cyanandrum* | x | x | x | x |
| Forb | *Polygonum macrophyllum* | x | x | - | x |
| Forb | *Polygonum runcinatum* | - | x | x | x |
| Forb | *Polystichum castaneum* | - | x | - | - |
| Forb | *Potentilla leuconota* | x | x | x | x |
| Forb | *Potentilla stenophylla var. emergens* | x | x | - | - |
| Forb | *Primula deflexa* | x | x | x | - |
| Forb | *Primula serratifolia* | x | - | x | - |
| Forb | *Prunella hispida* | - | x | x | x |
| Forb | *Pyrethrum tatsienense* | x | x | - | - |
| Forb | *Ranunculus brotherusii var. tanguticus* | x | x | x | x |
| Forb | *Rheum pumilum* | x | - | - | - |
| Forb | *Rhodiola fastigiata* | x | x | - | - |
| Forb | *Rhodiola yunnanensis* | x | x | x | x |
| Forb | *Rhododendron websterianum* | x | - | - | - |
| Forb | *Rumex nepalensis* | - | x | - | x |
| Forb | *Sagina japonica* | x | x | x | x |
| Forb | *Saussurea ceterach* | x | x | x | - |
| Forb | *Saussurea globosa* | x | x | - | - |
| Forb | *Saussurea graminea* | x | - | x | x |
| Forb | *Saussurea pachyneura* | x | - | - | - |
| Forb | *Saussurea stella* | x | x | - | - |
| Forb | *Saxifraga sp.* | x | - | - | - |
| Forb | *Sedum triactina* | - | x | - | - |
| Forb | *Sibbaldia pentaphylla* | x | - | - | - |
| Forb | *Soroseris hookeriana* | x | x | - | - |
| Forb | *Spiraea japonica* | - | - | x | - |
| Forb | *Stellaria uliginosa* | x | - | x | - |
| Forb | *Swertia macrosperma* | x | x | x | x |
| Forb | *Thalictrum javanicum* | - | - | - | x |
| Forb | *Trifolium repens* | - | - | x | x |
| Forb | *Veronica filipes* | x | - | - | - |
| Forb | *Veronica szechuanica* | x | x | x | x |
| Forb | *Viola biflora var. rockiana* | x | x | x | - |
| Forb | *Viola szetschwanensis* | - | x | x | - |
| Forb | *Youngia racemifera* | - | x | x | - |
| Graminoid | *Agrostis nervosa* | x | x | x | x |
| Graminoid | *Carex nubigena* | - | - | x | x |
| Graminoid | *Carex spp* | x | x | x | x |
| Graminoid | *Fargesia spathacea* | - | x | x | - |
| Graminoid | *Fargesia spp.* | - | x | x | - |
| Graminoid | *Festuca spp* | x | x | x | x |
| Graminoid | *Juncus allioides* | - | x | x | - |
| Graminoid | *Juncus himalensis* | x | x | x | - |
| Graminoid | *Juncus leucanthus* | x | x | x | - |
| Graminoid | *Juncus prismatocarpus* | - | - | - | x |
| Graminoid | *Juncus setchuensis* | - | - | x | x |
| Graminoid | *Kobresia cercostachys* | x | - | - | - |
| Graminoid | *Kobresia pygmaea* | x | x | - | - |
| Graminoid | *Luzula spp* | x | x | x | - |
| Graminoid | *Poa spp* | x | x | x | x |
| Woody | *Berberis jamesiana* | x | x | - | - |
| Woody | *Potentilla fruticosa* | x | x | - | - |
| Woody | *Quercus aquifolioides* | - | x | - | - |
| Woody | *Salix brachista* | x | - | - | - |

**Table S3** Estimate, standard error and t statistics shown for models testing change in species richness, evenness, and proportion of graminoids along the elevation gradient, represented by mean summer temperature (left), and in OTCs and transplants, represented by 1.5 °C and 1.75 °C temperature contrasts. For the gradient approach, an intercept-only null model (No effect) and a model with mean summer temperature at each site (Effect) are presented. For the two experimental warming approaches (OTC and Transplant), three models are presented: one containing the experimental site (No effect), one containing experimental site and temperature contrast testing the overall effect across sites (Effect) and one with the interactions of experimental site and temperature contrast (Interaction).

| **Response** | **Approach** | **Model** | **Term** | **Estimate** | **Std. error** | **T statistics** |
| --- | --- | --- | --- | --- | --- | --- |
| Richness | Gradient | No effect | Intercept | 15.08 | 0.55 | 27.35 |
|  |  | Effect | Intercept | 19.1 | 0.71 | 26.85 |
|  |  |  | Alpine | -2.46 | 0.93 | -2.64 |
|  |  |  | Middle | -3.39 | 0.93 | -3.63 |
|  |  |  | Lowland | -9.1 | 0.93 | -9.77 |
|  | OTC | No effect | Intercept | 18.8 | 0.84 | 22.45 |
|  |  |  | Alpine | -2.44 | 1.1 | -2.23 |
|  |  |  | Middle | -4.01 | 1.1 | -3.66 |
|  |  |  | Lowland | -10.23 | 1.1 | -9.33 |
|  |  | Effect | Intercept | 19.95 | 0.82 | 24.23 |
|  |  |  | Contrast | -1.54 | 0.44 | -3.49 |
|  |  |  | Alpine | -2.44 | 0.99 | -2.47 |
|  |  |  | Middle | -4.01 | 0.99 | -4.06 |
|  |  |  | Lowland | -10.23 | 0.99 | -10.36 |
|  |  | Interaction | Intercept | 19 | 1.04 | 18.23 |
|  |  |  | Contrast | -0.27 | 0.98 | -0.27 |
|  |  |  | Alpine | -1 | 1.36 | -0.73 |
|  |  |  | Middle | -3.71 | 1.36 | -2.72 |
|  |  |  | Lowland | -8.43 | 1.36 | -6.18 |
|  |  |  | Contrast:Alpine | -1.92 | 1.29 | -1.5 |
|  |  |  | Contrast:Middle | -0.4 | 1.29 | -0.31 |
|  |  |  | Contrast:Lowland | -2.4 | 1.29 | -1.87 |
|  | Transplant | No effect | Intercept | 21 | 0.82 | 25.58 |
|  |  |  | Alpine | -2.21 | 1.12 | -1.98 |
|  |  |  | Middle | -6.71 | 1.12 | -6 |
|  |  | Effect | Intercept | 20.67 | 0.96 | 21.45 |
|  |  |  | Contrast | 0.34 | 0.51 | 0.66 |
|  |  |  | Alpine | -2.15 | 1.13 | -1.9 |
|  |  |  | Middle | -6.74 | 1.13 | -5.97 |
|  |  | Interaction | Intercept | 19 | 1.19 | 15.92 |
|  |  |  | Contrast | 2.08 | 0.95 | 2.19 |
|  |  |  | Alpine | -1 | 1.56 | -0.64 |
|  |  |  | Middle | -3.71 | 1.56 | -2.38 |
|  |  |  | Contrast:Alpine | -1.06 | 1.33 | -0.8 |
|  |  |  | Contrast:Middle | -3.05 | 1.17 | -2.6 |
| Evenness | Gradient | No effect | Intercept | 0.77 | 0.01 | 61.48 |
|  |  | Effect | Intercept | 0.82 | 0.03 | 31.33 |
|  |  |  | Alpine | -0.04 | 0.03 | -1.21 |
|  |  |  | Middle | -0.01 | 0.03 | -0.29 |
|  |  |  | Lowland | -0.11 | 0.03 | -3.17 |
|  | OTC | No effect | Intercept | 0.83 | 0.03 | 27.32 |
|  |  |  | Alpine | -0.05 | 0.04 | -1.35 |
|  |  |  | Middle | -0.04 | 0.04 | -1.08 |
|  |  |  | Lowland | -0.13 | 0.04 | -3.4 |
|  |  | Effect | Intercept | 0.86 | 0.03 | 27.46 |
|  |  |  | Contrast | -0.04 | 0.02 | -2.56 |
|  |  |  | Alpine | -0.05 | 0.04 | -1.43 |
|  |  |  | Middle | -0.04 | 0.04 | -1.14 |
|  |  |  | Lowland | -0.13 | 0.04 | -3.59 |
|  |  | Interaction | Intercept | 0.83 | 0.04 | 21.51 |
|  |  |  | Contrast | -0.01 | 0.04 | -0.27 |
|  |  |  | Alpine | -0.06 | 0.05 | -1.17 |
|  |  |  | Middle | -0.02 | 0.05 | -0.39 |
|  |  |  | Lowland | -0.06 | 0.05 | -1.17 |
|  |  |  | Contrast:Alpine | 0.01 | 0.05 | 0.17 |
|  |  |  | Contrast:Middle | -0.03 | 0.05 | -0.64 |
|  |  |  | Contrast:Lowland | -0.1 | 0.05 | -2.1 |
|  | Transplant | No effect | Intercept | 0.83 | 0.03 | 29.72 |
|  |  |  | Alpine | -0.06 | 0.04 | -1.69 |
|  |  |  | Middle | -0.1 | 0.04 | -2.59 |
|  |  | Effect | Intercept | 0.87 | 0.03 | 29.91 |
|  |  |  | Contrast | -0.05 | 0.02 | -3.1 |
|  |  |  | Alpine | -0.07 | 0.03 | -2.15 |
|  |  |  | Middle | -0.09 | 0.03 | -2.77 |
|  |  | Interaction | Intercept | 0.83 | 0.04 | 22.65 |
|  |  |  | Contrast | -0.01 | 0.03 | -0.34 |
|  |  |  | Alpine | -0.06 | 0.05 | -1.23 |
|  |  |  | Middle | -0.02 | 0.05 | -0.41 |
|  |  |  | Contrast:Alpine | -0.01 | 0.04 | -0.2 |
|  |  |  | Contrast:Middle | -0.08 | 0.04 | -2.07 |
| Proportion Graminoid | Gradient | No effect | Intercept | 0.32 | 0.02 | 19.57 |
|  |  | Effect | Intercept | 0.3 | 0.04 | 8.07 |
|  |  |  | Alpine | -0.02 | 0.05 | -0.31 |
|  |  |  | Middle | 0.05 | 0.05 | 0.92 |
|  |  |  | Lowland | 0.03 | 0.05 | 0.64 |
|  | OTC | No effect | Intercept | 0.34 | 0.05 | 6.62 |
|  |  |  | Alpine | -0.07 | 0.07 | -1.02 |
|  |  |  | Middle | 0.04 | 0.07 | 0.62 |
|  |  |  | Lowland | 0.01 | 0.07 | 0.19 |
|  |  | Effect | Intercept | 0.32 | 0.06 | 5.6 |
|  |  |  | Contrast | 0.04 | 0.03 | 1.23 |
|  |  |  | Alpine | -0.07 | 0.07 | -1.02 |
|  |  |  | Middle | 0.04 | 0.07 | 0.62 |
|  |  |  | Lowland | 0.01 | 0.07 | 0.2 |
|  |  | Interaction | Intercept | 0.28 | 0.07 | 3.9 |
|  |  |  | Contrast | 0.08 | 0.07 | 1.23 |
|  |  |  | Alpine | 0.01 | 0.09 | 0.06 |
|  |  |  | Middle | 0.02 | 0.09 | 0.2 |
|  |  |  | Lowland | 0.09 | 0.09 | 0.96 |
|  |  |  | Contrast:Alpine | -0.1 | 0.09 | -1.11 |
|  |  |  | Contrast:Middle | 0.03 | 0.09 | 0.35 |
|  |  |  | Contrast:Lowland | -0.1 | 0.09 | -1.16 |
|  | Transplant | No effect | Intercept | 0.29 | 0.05 | 5.71 |
|  |  |  | Alpine | 0.04 | 0.07 | 0.62 |
|  |  |  | Middle | 0.2 | 0.07 | 2.8 |
|  |  | Effect | Intercept | 0.19 | 0.05 | 3.76 |
|  |  |  | Contrast | 0.11 | 0.03 | 3.95 |
|  |  |  | Alpine | 0.06 | 0.06 | 1.07 |
|  |  |  | Middle | 0.19 | 0.06 | 3.18 |
|  |  | Interaction | Intercept | 0.28 | 0.06 | 4.6 |
|  |  |  | Contrast | 0.01 | 0.05 | 0.22 |
|  |  |  | Alpine | 0.01 | 0.08 | 0.07 |
|  |  |  | Middle | 0.02 | 0.08 | 0.24 |
|  |  |  | Contrast:Alpine | 0.05 | 0.07 | 0.76 |
|  |  |  | Contrast:Middle | 0.17 | 0.06 | 2.83 |

**Table S4** R squared, degrees of freedom (df), AIC value and residual degrees of freedom for the test change in species richness, evenness, and proportion of graminoids along the elevation gradient, represented by mean summer temperature (left), and in OTCs and transplants, represented by 1.5 °C and 1.75 °C temperature contrasts. For the gradient approach, an intercept-only null model (No effect) and a model with mean summer temperature at each site (Effect) are presented. For the two experimental warming approaches (OTC and Transplant), three models are presented: one containing the experimental site (No effect), one containing experimental site and temperature contrast testing the overall effect across sites (Effect) and one with the interactions of experimental site and temperature contrast (Interaction). Models in bold and marked with an asterisk have the lowest AIC values and are considered the best model.

| **Response** | **Experiment** | **Model** | **R squared** | **df** | **AIC** | **df residual** |
| --- | --- | --- | --- | --- | --- | --- |
| Richness | Gradient | No effect | 0 | 1 | 294.1 | 51 |
|  |  | **Effect*** | 0.698 | 4 | 237.7 | 48 |
|  | OTC | No effect | 0.683 | 4 | 254.7 | 48 |
|  |  | **Effect*** | 0.748 | 5 | 244.7 | 47 |
|  |  | Interaction | 0.775 | 8 | 244.9 | 44 |
|  | Transplant | No effect | 0.508 | 3 | 202.0 | 37 |
|  |  | Effect | 0.514 | 4 | 203.5 | 36 |
|  |  | **Interaction*** | 0.602 | 6 | 199.6 | 34 |
| Evenness | Gradient | No effect | 0 | 1 | -99.2 | 51 |
|  |  | **Effect*** | 0.223 | 4 | -106.3 | 48 |
|  | OTC | No effect | 0.212 | 4 | -90.6 | 48 |
|  |  | Effect | 0.309 | 5 | -95.3 | 47 |
|  |  | **Interaction*** | 0.407 | 8 | -97.4 | 44 |
|  | Transplant | No effect | 0.156 | 3 | -68.9 | 37 |
|  |  | Effect | 0.334 | 4 | -76.4 | 36 |
|  |  | **Interaction*** | 0.430 | 6 | -78.7 | 34 |
| Proportion Graminoid | Gradient | **No effect*** | 0 | 1 | -71.5 | 51 |
|  |  | Effect | 0.045 | 4 | -67.9 | 48 |
|  | OTC | **No effect*** | 0.067 | 4 | -34.4 | 48 |
|  |  | Effect | 0.096 | 5 | -34.0 | 47 |
|  |  | Interaction | 0.174 | 8 | -32.7 | 44 |
|  | Transplant | No effect | 0.195 | 3 | -20.1 | 37 |
|  |  | Effect | 0.438 | 4 | -32.5 | 36 |
|  |  | **Interaction*** | 0.558 | 6 | -38.0 | 34 |

**Table S5** Species scores from PRC analysis from the controls at the origin site and the two warming treatments. The colours code goes from negative (red) to positive (dark green) scores.

|  |  | **High alpine** | | **Alpine** | | **Middle** | | **Lowland** |
| --- | --- | --- | --- | --- | --- | --- | --- | --- |
| **Species** | **Family** | **OTC** | **Transplant** | **OTC** | **Transplant** | **OTC** | **Transplant** | **OTC** |
| *Allium prattii* | Liliaceae | 0.572 | 0.914 |  |  |  |  |  |
| *Anaphalis aureopunctata* | Asteraceae |  |  |  |  | -1.077 | 1.312 |  |
| *Androsace minor* | Primulaceae |  | -1.181 |  |  |  |  |  |
| *Artemisia flaccida* | Asteraceae |  |  | -0.723 |  |  | 0.787 |  |
| *Carpesium humile* | Asteraceae |  |  |  |  | -0.986 | 0.864 |  |
| *Clinopodium polycephalum* | Labiatae |  |  |  |  | -0.542 | -1.628 |  |
| *Cyananthus incanus* | Campanulaceae | 1.332 | -0.922 |  |  |  |  |  |
| *Epilobium fangii* | Onagraceae |  |  |  |  | -0.598 |  |  |
| *Fragaria orientalis* | Rosaceae |  |  | -0.55 | 0.685 | -4.731 |  |  |
| *Galearis spathulata* | Orchidaceae |  | -0.787 |  |  |  |  |  |
| *Galium spp* | Rubiaceae |  |  |  |  |  | -2.037 |  |
| *Geranium pylzowianum* | Geraniaceae |  | -0.622 | 1.454 | -0.859 | -2.172 | 3.575 |  |
| *Hemiphragma heterophyllum* | Scrophulariaceae |  |  | -0.63 |  | 0.665 | 1.432 |  |
| *Hypericum wightianum* | Guttiferae |  |  |  |  |  | 0.574 |  |
| *Ligusticum scapiforme* | Umbelliferae | 0.936 | -4.486 | 4.974 | 4.318 |  |  |  |
| *Parasenecio latipes* | Asteraceae |  |  | 1.04 | 0.796 |  |  |  |
| *Parnassia spp* | Parnassiaceae | -0.972 |  |  |  |  |  |  |
| *Pedicularis davidii* | Scrophulariaceae |  |  |  | -0.707 | 1.73 | 2.824 |  |
| *Pedicularis sima* | Scrophulariaceae |  |  |  | -0.662 |  |  |  |
| *Plantago asiatica* | Plantaginaceae |  |  |  |  |  | -0.846 | 0.926 |
| *Polygonum macrophyllum* | Polygonaceae | 2.079 | 2.907 |  | -0.627 |  |  |  |
| *Polygonum runcinatum* | Polygonaceae |  |  |  |  |  | -0.629 |  |
| *Persicaria vivipara* | Polygonaceae |  | -3.41 | -1.866 | 5.529 |  |  |  |
| *Potentilla leuconota* | Rosaceae |  |  |  | -0.862 | 3.221 | 2.823 | -5.697 |
| *Potentilla stenophylla var. emergens* | Rosaceae | 4.168 | 1.067 |  |  |  |  |  |
| *Prunella hispida* | Labiatae |  |  |  |  |  | 0.56 |  |
| *Saussurea ceterach* | Asteraceae |  | -1.236 |  | -0.805 |  |  |  |
| *Saussurea globosa* | Asteraceae |  | -0.588 | -2.082 | 1.57 |  |  |  |
| *Saussurea graminea* | Asteraceae | 0.846 |  |  |  |  |  |  |
| *Sibbaldia pentaphylla* | Rosaceae |  | -0.812 |  |  |  |  |  |
| *Maianthemum japonicum* | Liliaceae |  |  | -0.559 |  |  |  |  |
| *Soroseris hookeriana* | Asteraceae | -0.784 |  | -1.467 | 1.885 |  |  |  |
| *Swertia macrosperma* | Gentiana |  |  |  | -0.618 |  | 0.635 |  |
| *Trifolium repens* | Leguminosae |  |  |  |  |  | -2.403 | 5.397 |
| *Veronica szechuanica* | Plantaginaceae |  |  |  | -0.609 |  |  |  |
| *Viola biflora var. rockiana* | Violaceae | 3.627 | 2.179 |  |  |  |  |  |
| *Youngia racemifera* | Asteraceae |  |  |  |  | -0.616 | 2.267 |  |
